# RiboTag translatomic profiling of *Drosophila* oenocytes under aging and induced oxidative stress

**DOI:** 10.1186/s12864-018-5404-4

**Published:** 2019-01-16

**Authors:** Kerui Huang, Wenhao Chen, Fang Zhu, Patrick Wai-Lun Li, Pankaj Kapahi, Hua Bai

**Affiliations:** 1 Department of Genetics, Development, and Cell Biology, Iowa State University, Ames, IA 50011 USA; 2 Department of Electrical and Computer Engineering, Iowa State University, Ames, IA 50011 USA; 3 Department of Entomology, Pennsylvania State University, University Park, PA 16802 USA; 4 Buck Institute for Research on Aging, Novato, CA 94945 USA

**Keywords:** Oenocyte, Fat body, Liver, Aging, Ribosomal profiling, Peroxisome, Fatty acid beta-oxidation, Ras/MAPK signaling

## Abstract

**Background:**

Aging is accompanied with loss of tissue homeostasis and accumulation of cellular damages. As one of the important metabolic centers, liver shows age-related dysregulation of lipid metabolism, impaired detoxification pathway, increased inflammation and oxidative stress response. However, the mechanisms for these age-related changes still remain unclear. In the fruit fly, *Drosophila melanogaster*, liver-like functions are controlled by two distinct tissues, fat body and oenocytes. Compared to fat body, little is known about how oenocytes age and what are their roles in aging regulation. To characterize age- and stress-regulated gene expression in oenocytes, we performed cell-type-specific ribosome profiling (RiboTag) to examine the impacts of aging and oxidative stress on oenocyte translatome in *Drosophila*.

**Results:**

We show that aging and oxidant paraquat significantly increased the levels of reactive oxygen species (ROS) in adult oenocytes of *Drosophila*, and aged oenocytes exhibited reduced sensitivity to paraquat treatment. Through RiboTag sequencing, we identified 3324 and 949 differentially expressed genes in oenocytes under aging and paraquat treatment, respectively. Aging and paraquat exhibit both shared and distinct regulations on oenocyte translatome. Among all age-regulated genes, oxidative phosphorylation, ribosome, proteasome, fatty acid metabolism, and cytochrome P450 pathways were down-regulated, whereas DNA replication and immune response pathways were up-regulated. In addition, most of the peroxisomal genes were down-regulated in aged oenocytes, including genes involved in peroxisomal biogenesis factors and fatty acid beta-oxidation. Many age-related mRNA translational changes in oenocytes are similar to aged mammalian liver, such as up-regulation of innate immune response and Ras/MAPK signaling pathway and down-regulation of peroxisome and fatty acid metabolism. Furthermore, oenocytes highly expressed genes involving in liver-like processes (e.g., ketogenesis).

**Conclusions:**

Our oenocyte-specific translatome analysis identified many genes and pathways that are shared between *Drosophila* oenocytes and mammalian liver, highlighting the molecular and functional similarities between the two tissues. Many of these genes were altered in both oenocytes and liver during aging. Thus, our translatome analysis provide important genomic resource for future dissection of oenocyte function and its role in lipid metabolism, stress response and aging regulation.

**Electronic supplementary material:**

The online version of this article (10.1186/s12864-018-5404-4) contains supplementary material, which is available to authorized users.

## Background

Aging is the major risk factor for many chronic diseases [1]. The prevalence of liver diseases, such as non-alcoholic fatty liver disease (NAFLD), increase dramatically in the elderly [2, 3]. It is known that aging is associated with alterations of hepatic structure, physiology and function [4]. For example, aged liver shows reduced blood flow, loss of regenerative capacity, decreases in detoxification and microsomal proteins synthesis, increases in polyploidy, oxidative stress and mitochondrial damage [5]. Additionally, the metabolism for low-density lipoprotein cholesterol decreases by 35% [3]. Age-related increases in neutral fat levels and high-density lipoprotein cholesterol predispose aged liver to NAFLD and other liver diseases. Accumulated evidence suggests that age-related decline of liver function can be attributed to increased reactive oxygen species (ROS) production, DNA damage, activation of p300-C/EBP-dependent neutral fat synthesis [6], decreases in autophagy, increases in inflammatory responses [7, 8], and activation of nuclear factor-κB (NF-κB) pathway [4, 9]. Despite the genetic and functional analysis of liver aging and liver diseases, only a few studies have looked at the global transcriptional and translational changes during liver aging [10–13].

Similar to mammals, the fruit fly (*Drosophila melanogaster*, hereafter as *Drosophila*) also shows age-dependent decline of tissue function and loss of homeostasis (reviewed in [14]). In *Drosophila*, liver-like functions are shared by two distinct tissues, fat body and oenocytes [15]. Fat body is the main tissue for energy storage in insects, and it plays a key role in metabolism, nutrition sensing, growth and immunity (reviewed in [16]). Fat body has also been implicated in the regulation of organismal aging [17]. Many longevity pathways act on fat body to control lifespan [18–20]. Compared to fat body, little is known about how oenocytes age and what is the role of oenocytes in aging regulation. Oenocytes are specialized hepatocyte-like cells responsible for energy metabolism, biosynthesis of cuticular hydrocarbon and pheromone ([15, 21], reviewed in [22, 23]). Oenocytes coordinate with fat body in mobilizing lipid storage upon nutrient deprivation [15, 24, 25]. Recent studies in the yellow fever mosquito *Aedes aegypti* showed that pupal oenocytes highly express cytochrome P450 genes, suggesting an important role of oenocytes in detoxification [26]. Despite its roles in lipid metabolism and wax production, we know very little about oenocyte’s other physiological functions, including its role in the regulation of aging and longevity. It is known that aging oenocytes undergo dramatic morphological changes (e.g., increases in cell size and pigmented granules [27]) and exhibit dysregulation of mitochondrial chaperone *Hsp22* [28]. Mitochondrial ROS production increases with age or under acute oxidative stress (induced by oxidants like paraquat, PQ) [29]. To date, transcriptional characterization of oenocyte aging has not been previously performed.

Here, we utilized RiboTag technique [30] to profile changes in ribosome-associated transcripts (translatome) in *Drosophila* oenocyte during aging and PQ-induced oxidative stress. We show that aging and PQ exhibit common and distinct regulation on adult oenocyte translatome. Gene ontology (GO) and gene set enrichment analysis (GSEA) revealed that ribosome, proteasome, peroxisome, xenobiotic metabolism, fatty acid metabolism, and DNA replication pathways were altered under aging and oxidative stress. Comparing tissue-specific transcriptomes and proteomes further revealed that oenocytes were enriched with genes involved in liver-like functions (e.g., ketogenesis). Aging oenocytes also shared many molecular signatures with aging liver. Taken together, our translatome analysis revealed a conserved molecular mechanism underlying oenocyte and liver aging. Our study will offer new opportunities for future dissection of novel roles of oenocytes in lipid metabolism, stress response, and aging control.

## Results

### Characterization of age-related changes in ROS production in *Drosophila* oenocytes

**Fig. 1:**
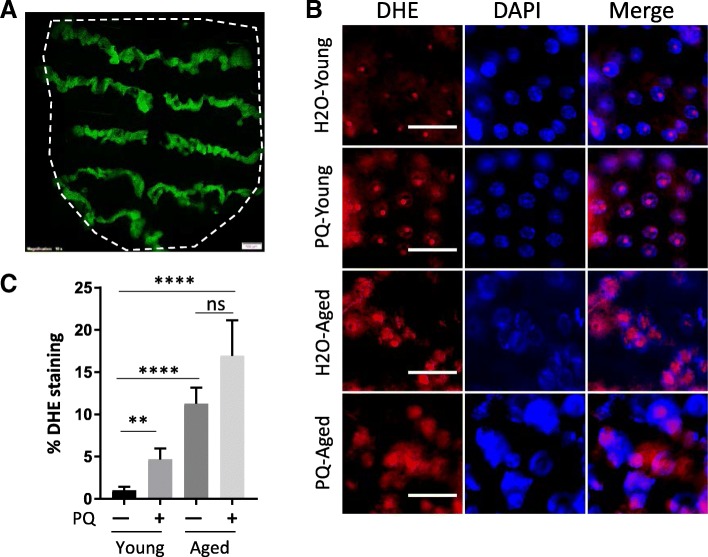
Characterization of age-related changes in ROS production in *Drosophila* oenocytes. **a** Fluorescent image of GFP-labeled oenocytes from one fly dorsal abdomen dissected from a *PromE-Gal4; UAS-CD8::GFP* female. The dashed line indicates the location of the fly abdomen. Scale bar: 100 μm. **b** ROS levels indicated by DHE staining in female oenocytes under aging and paraquat (PQ) treatment. Young: 10-day-old, Aged: 30-day-old. DAPI stains for nuclei. Scale bar: 10 μm. **c** Quantification of DHE staining from Panel (**b**). One-way ANOVA (**** *p* < 0.0001, ** *p* < 0.01, ns = not significant ). *N* = 5

In *Drosophila*, larval and adult oenocytes exhibit distinct morphological characteristics [22]. Larval oenocytes are clustering along the lateral body wall [15], while adult oenocytes (used in the present study) appear as segmental dorsal stripes and ventral clusters nearby the abdominal cuticle (Fig. 1a). As oxidative stress is commonly observed in aging tissue, we first examined the age-related changes in ROS production in adult oenocytes. As shown in Fig. 1b and c, both aging and PQ (an oxidative stress inducer) significantly increased ROS levels in adult oenocytes. Increases in cell and nuclear sizes were also seen in aged oenocytes (Fig. 1b, Additional file 1: Figure S1). In the present study, oenocytes were dissected from two ages, 10 days (young) and 30 days (middle age). Middle age was used because many epigenetic and transcriptional changes have been previously observed in the midlife [31–33]. Since elevated ROS levels were already apparent at middle age, a comparison between young and middle age will allow us to capture the early-onset age-related changes in adult oenocytes. Additionally, we noticed that young oenocytes showed much higher induction of ROS under PQ treatment than the oenocytes from middle age (Fig. 1c), suggesting the response to oxidative stress was altered in aged oenocytes.

### Oenocyte-specific translatomic profiling through RiboTag sequencing

**Fig. 2:**
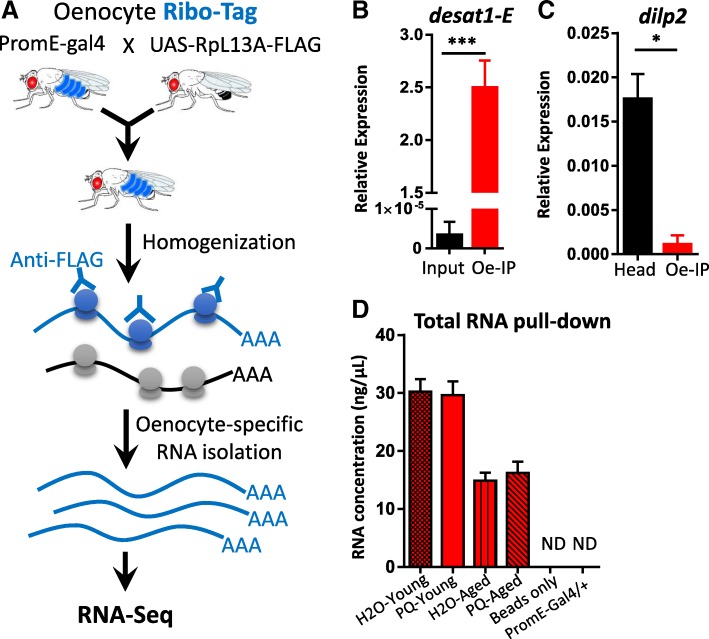
Oenocyte-specific translatomic profiling through RiboTag sequencing. **a** Schematic diagram showing RiboTag procedures. FLAG-tagged ribosomal protein RpL13A was first ectopically expressed in oenocytes. Translating RNAs were immunoprecipitated using anti-FLAG antibodies. RNAs were further purified and used in RNA-seq analysis. **b** Oenocyte-specific transcript *desat1-E* highly expressed in anti-FLAG immunoprecipitated sample (IP) compared to the input (whole body lysate). **c** The transcripts of brain-specific gene *Dilp2* was enriched in head samples compared to oenocyte RiboTag samples. One-way ANOVA ( *** *p* < 0.001, * *p* < 0.05, ns = not significant). *N* = 3. **d** RNA concentrations of various immunoprecipitated samples. ND: Not detected. 200 female flies were used in each condition. Three biological replicates per condition

Besides their roles in metabolic homeostasis, hydrocarbon and pheromone production (reviewed in [22]), the role of oenocytes in aging regulation has not been carefully examined. Characterization of age-related translatomic changes in oenocytes is an important step toward our understanding of oenocyte aging. To date, only a few oenocyte transcriptome analyses have been reported [24, 26]. Most of these studies used dissected oenocytes, which often have issues with tissue cross-contamination. To overcome this issue, we performed an oenocyte-specific RiboTag analysis. In the analysis, oenocyte-specific driver *PromE-Gal4* was used to drive the expression of FLAG-tagged *RpL13A*. According to RNA-seq database (at Flybase.org) and a recent ribosomal proteome analysis [34], RpL13A is one of the highly and ubiquitously expressed components in *Drosophila* large ribosomal subunit. Our experimental design facilitates the enrichment of oenocyte-specific ribosome-associated mRNAs and translatomic profiling (Fig. 2a). To verify the efficiency and specificity of our RiboTag profiling, we performed a qRT-PCR analysis to measure the expression of *Desaturase 1 (Desat1). Desat1* is a transmembrane fatty acid desaturase and its E isoform (*desat1-E*) was known to be specifically expressed in female oenocytes [21]. We found that the expression of *desat1-E* was much higher in anti-FLAG immunoprecipitated sample (oenocytes) compared to the input (whole body), suggesting that our RiboTag approach can effectively detect the gene expression from adult oenocytes (Fig. 2b).

To confirm the specificity of the RiboTag analysis, we measured the expression of a brain-specific gene, *insulin-like peptide 2* (*Dilp2*), and found that *Dilp2* expression in oenocyte RiboTag samples was very low compared to the head samples (Fig. 2c). Thus our RiboTag analysis has very little contamination from other tissues (such as brain). We also set up two control experiments to test the specificity of the reagents used in our pull-down assay: 1) Immunoprecipitation of *PromE > RpL13A-FLAG* expressing females using only protein G magnetic beads without adding FLAG antibody. 2) Immunoprecipitation of *PromE-gal4* flies using both Protein G magnetic beads and FLAG antibody. No detectable RNAs were pulled down from the two control groups, suggesting there is none or very little non-specific binding from FLAG antibodies or protein G magnetic beads during the immunoprecipitation (Fig. 2d). Notably, the total RNA pulled down from aged samples were less than those from young oenocytes. This is probably due to age-related decreases in general transcription and translation, because the *PromE-gal4* driver activity remained the same during aging (Additional file 1: Figure S1). Due to the variation in RNA quantity among different samples, we used equal amount of RNAs for all library construction. To examine age- and stress-related translatomic changes in *Drosophila* oenocytes, we performed RiboTag sequencing on four different experimental groups: H2O-Young, PQ-Young, H2O-Aged, PQ-Aged (see Methods for more details). Female flies were used in the present study, because previous studies showed that *PromE-gal4* drives expression in testis (additional to oenocytes) in male flies [21].

### Differential gene expression (DGE) analysis reveals common and distinct mRNA translational regulation by aging and oxidative stress

**Fig. 3:**
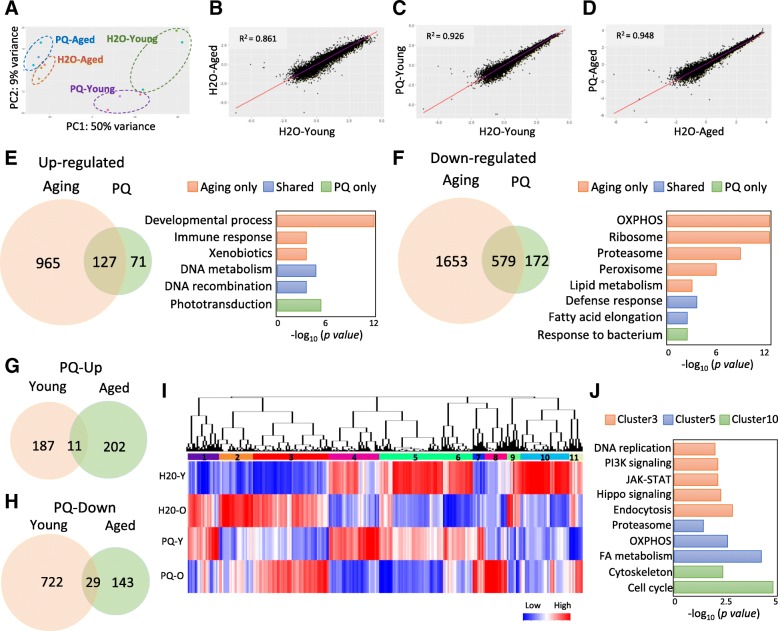
Differential gene expression analysis reveals common and distinct translational regulation by aging and oxidative stress. **a** Principal component analysis (PCA) on four oenocyte translatomes. **b-d** Correlation analysis on the gene expression between H2O-Young and H2O-Aged; H2O-Young and PQ-Young; H2O-Aged and PQ-Aged. Log_10_ (FPKM) was used in the analysis **e-f** Venn diagram and GO terms for the genes commonly and differentially regulated by aging and paraquat. **g-h** Venn diagram and GO terms for the genes commonly and differentially regulated by paraquat at young and old ages. **i** Hierarchy clustering analysis on oenocyte translatome. **j** Gene ontology analysis on cluster 3, 5, 10 in panel (**i**). OXPHOS: oxidative phosphorylation. FA: fatty acid

Using Illumina sequencing (HiSeq 3000, single-end, a read length of 50 base pair), we obtained a total of 402 million reads from 12 library samples. On average, 82.43% of unique reads were mapped to annotated *Drosophila* reference genome (Release 6). To visualize how gene expression varies under different conditions, we performed principal component analysis (PCA) on the fragments per kilobase million (FPKM) reads. The first component accounts for 50% of the variance and the second component accounts for 9% of variance (Fig. 3a). The PCA analysis showed that three replicates of each condition cluster together, except for one of the H2O-young samples. Two age groups were also well separated. Interestingly, there was a reduced variation between H2O and paraquat treatment in aged oenocytes compared to the young ones (Fig. 3a).

DGE analysis was performed using Cufflinks and Cuffdiff tools (fold change ≥2, FDR adjusted *p*-value ≤0.05, only protein-coding genes were analyzed). To compare the impacts of aging and oxidative stress on mRNA translational changes in adult oenocytes, we first performed correlation analysis using Log-transformed FPKM reads from all four groups. The coefficient of determination (R^2^) was 0.861 between H2O-aged and H2O-young groups (Fig. 3b), 0.926 between H2O-young and PQ-young (Fig. 3c), 0.948 between PQ-aged and H2O-aged (Fig. 3d). Aging induced a bigger mRNA translational shift compared to paraquat treatment. Although the change of R^2^ was relatively small, the total number of age-regulated genes was much higher than that under paraquat treatment (Fig. 3e and f). Thus, both PCA and correlation analyses suggest that aging and paraquat exhibit different impacts on oenocyte translatome.

DGE analysis identified 3324 genes that were differentially expressed during oenocyte aging (1092 up-regulated and 2232 down-regulated), while 949 genes (198 up-regulated and 751 down-regulated) were regulated by paraquat treatment at young ages (Fig. 3e and f, Additional file 2: List 1–4). About 706 DEGs were commonly regulated by aging and paraquat (127 up-regulated and 579 down-regulated) (Additional file 2: List 5–6). The genes commonly up-regulated by aging and PQ were involved in DNA metabolism and DNA recombination (Fig. 3e), while those commonly down-regulated genes were involved in defense response and fatty acid elongation (Fig. 3f, Additional file 3: List 1–4).

Besides common translatomic regulation between aging and oxidative stress, many genes were differentially regulated between the two processes. A total of 2618 genes (965 up-regulated and 1653 down-regulated) were only regulated by aging (Fig. 3e and f, Additional file 2: List 5–6). Genes up-regulated in aged oenocytes were enriched in several Gene ontology (GO) terms, including developmental process, immune response, and metabolism of xenobiotics (Additional file 3: List 5). The down-regulated genes are enriched in oxidative phosphorylation, ribosome, proteasome, peroxisome, and fatty acid metabolism (Additional file 3: List 6). About 243 genes (71 up-regulated and 172 down-regulated) were only regulated by paraquat treatment at young ages. These genes are enriched for biological processes like phototransduction and response to bacterium (Additional file 3: List 7–8).

It is known that stress tolerance declines with age [35], which can be caused by impaired transcriptional regulation of stress signaling pathways [36]. Our translatome analysis showed that the total number of PQ-regulated genes decreased with aging (Fig. 3g and h). About 949 genes were differentially expressed under paraquat treatment at young ages (198 up-regulated and 751 down-regulated), while only 385 genes were differentially expressed at middle ages (213 up-regulated and 172 down-regulated) (Additional file 2: List 7–8). In addition, paraquat treatment targeted a different sets of the biological processes and signaling pathways between young and middle ages (Fig. 3g and h). In young oenocytes, paraquat up-regulated pathways like response to DNA metabolism and DNA recombination, while down-regulating immune response, fatty acid biosynthesis, and fatty acid elongation (Additional file 3: List 9–10). In contrast, different sets of pathways were up-regulated by paraquat at middle ages, such as pheromone binding and cation channel activity (Additional file 3: List 11–12).

Next, we performed hierarchical clustering analysis and identified 11 distinct clusters among four groups (Fig. 3i). Among 11 clusters, cluster 3, cluster 5, and cluster 10 are three major clusters. Cluster 3 includes genes that were strongly up-regulated by aging but not PQ treatment. Gene ontology analysis showed that cluster 3 was enriched with genes in endocytosis, hippo, JAK-STAT, fanconi anemia pathway, phosphatidylinositol signaling, and DNA replication (Fig. 3j). Cluster 5 consisted of genes down-regulated by aging (less impacted by PQ), and was enriched in fatty acid metabolism, oxidative phosphorylation, and proteasome (Fig. 3j). In addition, clusters 10 included genes that were down-regulated in both aged and PQ-treated oenocytes, and was enriched in cell cycle and cytoskeleton organization (Fig. 3j). Taken together, our RiboTag analysis revealed common and distinct translatomic changes under aging and oxidative stress in adult oenocytes.

### Gene set enrichment analysis (GSEA) reveals key pathways that are up- and down-regulated in aged oenocytes

**Fig. 4:**
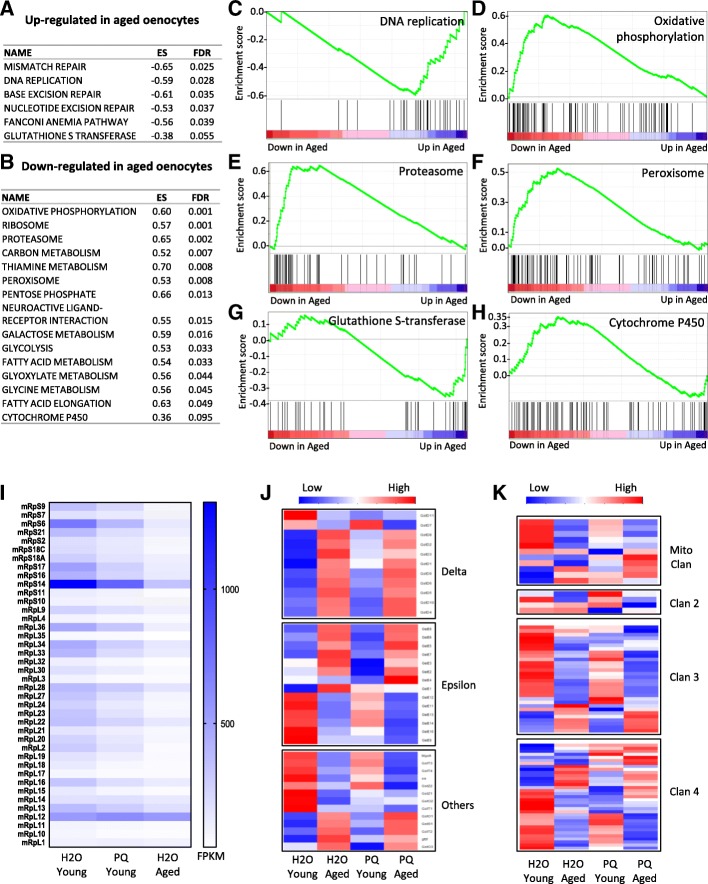
GSEA analysis revealed up- and down-regulated pathways under aging. **a** List of the pathways up-regulated in aged oenocytes. **b** List of the pathways down-regulated in aged oenocytes. ES: Enrichment score. **c-h** GSEA enrichment profiles of six pathways: DNA replication, oxidative phosphorylation, proteasome, peroxisome, glutathione S-transferase, cytochrome P450. **i-k** Heatmaps for mitochondrial ribosomal subunits, glutathione S-transferase, cytochrome P450

To further characterize oenocyte-specific signaling pathways that were regulated by aging and oxidative stress, we performed gene set enrichment analysis (GSEA) using a collection of pre-defined gene sets retrieved from Kyoto Encyclopedia of Genes and Genomes (KEGG) database. Through GSEA, we discovered five pathways within which genes were up-regulated with age (FDR *q*-value< 0.05) (Fig. 4a and c, Additional file 3: List 13). They are mismatch repair, DNA replication, base excision repair, nucleotide excision repair, and fanconi anemia pathways. These pathways were tightly related to the cellular responses to DNA replication stress, suggesting a possible increased DNA replication stress during oenocyte aging. Several key players in DNA replication stress response were up-regulated aged oenocytes, such as ATR/mei-41 (ATM- and Rad3-related kinase) and TopBP1/mus101 (DNA topoisomerase 2-binding protein 1).

On the other hand, GSEA analysis revealed 14 pathways that were significantly down-regulated during aging, such as oxidative phosphorylation, ribosome, proteasome, and peroxisome (Fig. 4b, d, e, f, Additional file 3: List 13). These results suggest that the functions of many key cellular organelles/components (e.g., mitochondria and peroxisome) were impaired in aged oenocytes. In aged oenocytes, we found that the key components of all five complexes in mitochondrial electron transport chain were down-regulated, such as NADH dehydrogenase subunits (e.g., *ND-13, ND-15, ND-30, ND-B8*), succinate dehydrogenase (e.g., *SdhC, SdhD*), cytochrome bc1 complex (e.g., *Cyt-c1, UQCR-14, UQCR-C2, UQCR-Q, ox*), cytochrome c oxidase subunits (e.g., *COX4, COX5A, COX5B*), and ATP synthase subunits (e.g., *ATPsynB, ATPsynD, ATPsynF, ATPsynO*) (Additional file 2: List 2). Interestingly, we found that aging down-regulated many mitochondrial ribosomal subunit genes (44 out of 72 annotated mitochondrial ribosomal proteins) (Fig. 4i) (Additional file 2: List 2). Lastly, we observed an age-related decrease in the expression proteasome subunit genes. These include 20S protein subunits (e.g., *Prosalpha2, Prosalpha3, Prosbeta1, Prosbeta2, Prosbeta3*), and 19S regulatory cap subunits (e.g., *Rpn1, Rpn11, Rpn12, Rpt1, Rpt2, Rpt3*) (Additional file 2: List 2).

Reduced xenobiotic metabolism is one of the hallmarks of liver aging [37]. Xenobiotics metabolism (or detoxification) consists of three major phases [38]. The Phase I and II enzymes represent the most abundant classes of detoxification system, including cytochrome P450 (CYPs) and glutathione S-transferases (GSTs). Interestingly, our GSEA analysis revealed distinct expression patterns for these two detoxification enzyme families. We found that almost all GSTs in Delta class were up-regulated under aging, while other classes showed mixed expression patterns (Fig. 4j, Additional file 2: List 9). The microsomal glutathione S-transferase (*Mgstl*), one of the highly enriched oenocyte genes, was significantly down-regulated during oenocyte aging (Additional file 2: List 9).

On the other hand, most of the cytochrome P450 genes were down-regulated in aged oenocytes (Fig. 4h and k). Many of the down-regulated CYPs have been previously linked to insecticide resistance or xenobiotic metabolism, such as *Cyp6a8, Cyp6a21, Cyp308a1, Cyp12a4, Cyp6a2, Cyp6w1, and Cyp313a1* (Additional file 2: List 10). Besides metabolizing exogenous chemicals, several CYPs catalyze endogenous metabolites and play key roles steroid hormone biosynthesis and fatty acid metabolism. For example, *Cyp4g1* is a key CYP gene involved in cuticular hydrocarbon biosynthesis [39] and triglyceride metabolism [15]. The expression of *Cyp4g1* was decreased in aged oenocytes (Additional file 2: List 10). About eight CYPs (also known as the Halloween genes) in *Drosophila* are known to regulate ecdysteroid metabolism. Two of them, *Cyp306a1* (*Phantom*) and *Cyp315a1* (*Shadow*), were highly expressed in oenocytes (32-fold and 12.5-fold enriched respectively) (Additional file 1: Figure S2). During oenocyte aging, *Phantom* was down-regulated, whereas *Shadow* was up-regulated (Additional file 2: List 10).

### Peroxisome pathway is deregulated in aged oenocytes

**Fig. 5:**
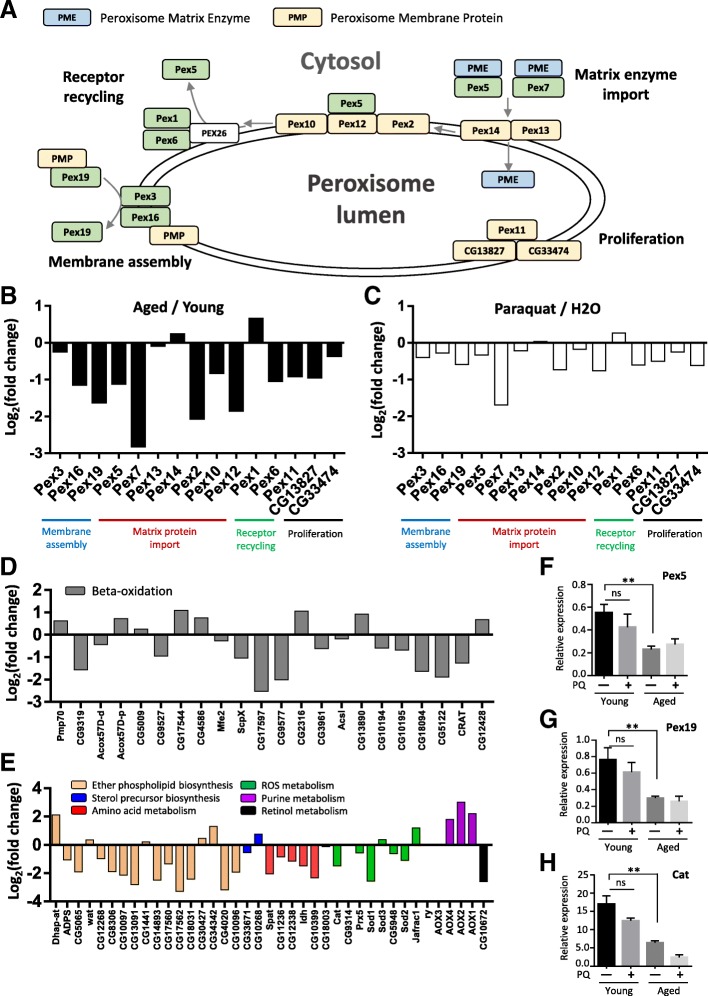
Peroxisome pathway is translationally deregulated in aged oenocytes. **a** Schematic diagram showing peroxisome pathway and the role of each peroxin (PEX) genes. **b** and **c** Log_2_ (fold change) of the expression of PEX genes under aging and paraquat treatment, based on oenocyte RiboTag sequencing results. **d** and **e** Log_2_ (fold change) of the expression of genes involved in other peroxisome functions during oenocyte aging. **f-h** qRT-PCR verification of three peroxisome genes (*Pex5, Pex19, Cat*). One-way ANOVA (** *p* < 0.01, ns = not significant). *N* = 3

Recent studies suggest that peroxisome protein import is impaired during aging [40]. Our GSEA analysis revealed that except for *Pex1* (up-regulated), most of the genes involved in peroxisome biogenesis (also called peroxin, *PEX*) were down-regulated in aged oenocytes (Figs. 4f and 5a, Additional file 2: List 11). Out of 16 peroxin genes, five of them showed significant down-regulation during aging (fold change ≥2, FDR adjusted *p*-value ≤0.05). They are matrix enzyme import components (*Pex5, Pex12*), receptor recycling (*Pex6*), and membrane assembly components (*Pex16, Pex19*) (Fig. 5a and b). In addition, most of the *PEX* genes were also down-regulated by paraquat treatment, but to a less extend comparing to aging (Fig. 5c).

Besides peroxisome biogenesis, genes involved in other peroxisomal functions were also down-regulated during oenocyte aging (Fig. 5d and e, Additional file 2: List 11). These functions include fatty acid beta-oxidation, ether phospholipid biosynthesis, amino acid metabolism, ROS metabolism, purine metabolism, and retinol metabolism. Several beta-oxidation genes showed significantly decreased expression, including sterol carrier protein X-related thiolases (*ScpX and CG17597*), enoyl-CoA hydratase (*ECH/CG9577*), carnitine O-acetyl-transferases (*CRAT and CG5122*), and nudix hydrolases (*CG10194, CG10195, CG18094*) (Fig. 5d). Consistently, hepatocyte nuclear factor 4 (*HNF4*), the major regulator for mitochondrial and peroxisomal beta-oxidation, was significantly down-regulated under aging and paraquat (Additional file 2: List 2). On the other hand, a few other beta-oxidative genes were up-regulated in aged oenocyte, such as ABC transporters (*Pmp70, CG2316*) that are responsible for transporting long-chain fatty acids into peroxisome, delta3-delta2-enoyl-CoA isomerase (*PECI/CG13890*), carnitine O-octanoyltransferase (*CROT/CG12428*). Acyl-CoA oxidases (*Acox*) that are involved in the first step of beta-oxidation showed mixed expression pattern (Fig. 5d).

Consistent with increased ROS production during oenocyte aging, most of the genes regulating peroxisomal ROS metabolism were down-regulated in aged oenocytes, such as *catalase* (*Cat*), *superoxide dismutase 1* (*SOD1*), *peroxiredoxin 5* (*Prx5*). Although the majority of ether phospholipid synthesis genes (e.g., fatty acyl-CoA reductase, *FAR*) were down-regulated, there are a few genes that showed up-regulation during aging, such as dihydroxyacetone phosphate acyltransferase (*DHAPAT* or *Dhap-at*), the key enzyme for the production of acyl-DHAP (the obligate precursor of ether lipids). Additionally, three aldehyde oxidases (*Aox1, Aox2, Aox4*) in purine metabolism were up-regulated (Fig. 5e).

To verify our RiboTag sequencing results, we performed qRT-PCR analysis on three selected peroxisome genes, *Pex5*, *Pex19*, and *Cat*. Consistent with RNA-Seq results, qRT-PCR showed that all three genes were significantly down-regulated in aged oenocytes (Fig. 5f-h).

### Ketogenesis, fatty acid elongation, and peroxisome pathways are enriched in both oenocytes and liver

Fat body, but not oenocytes, is a long-established tissue model to study liver- and adipose-like functions in *Drosophila* [41]. Although hepatocyte-like functions (e.g., steatosis) have been previously observed in oenocytes [15], it remains unclear whether fat body and oenocytes each perform different aspects of liver-like functions in *Drosophila*. To address this question, we performed a tissue-specific gene expression comparison among *Drosophila* oenocytes, fat body, and human liver (Table 1). Because the translatome of *Drosophila* fat body is currently not available, we used transcriptome data in the analysis, despite there are some caveats for such translatome and transcriptome comparison.

**Table 1:** Comparison of the GO terms enriched in oenocyte, fat body and liver

GO	Oenocyte	Fat body	Liver
Biological Process	Fatty acid biosynthesis Fatty acid elongation Oxidation reduction Immune response Protein catabolism	Carboxylic acid metabolism Immune response Amino acid metabolism	Lipid metabolism Long-chain fatty acid metabolism Oxidation reduction Immune response Xenobiotic metabolism Bile acid metabolism
Molecular Function	Oxidoreductase activity Endopeptidase activity Fatty acid synthase activity Fatty acid elongase activity Peptidoglycan binding	Oxidoreductase activity Metalloendopeptidase activity Heme binding	Oxidoreductase activity Serine-type peptidase activity Lipid binding Heme binding
Cellular Component	Peroxisome Proteasome	Extracellular region	Peroxisome Extracellular region Endoplasmic reticulum
KEGG Pathway	Metabolism of xenobiotics Peroxisome Proteasome Ketone body metabolism Unsaturated fatty acid synthesis	Glycine, serine and threonine metabolism Arginine and proline metabolism	Metabolism of xenobiotics Peroxisome Bile secretion PPAR signaling pathway Retinol metabolism Biosynthesis of amino acids
Protein Domain	ELO family Proteasome subunit	Peptidase S1, M13 EGF-like domain	Serpin family Cytochrome P450

We first identified genes that were enriched in adult oenocytes by comparing our oenocyte RiboTag data (H2O-Young group) with previously published whole body transcriptome data (Additional file 2: List 12). Fat body-enriched genes were identified based on *Drosophila* tissue transcriptome database, FlyAtlas [42, 43] (Additional file 2: List 13). The genes with more than 5-fold higher expression in oenocytes (or fat body) comparing to whole body are defined as oenocyte-enriched (or fat body-enriched) genes. A total of 423 oenocyte-enriched genes and 544 fat body-enriched genes were identified through tissue translatome/transcriptome comparison (Additional file 1: Figure S3). A recent study showed that *Drosophila* oenocytes express many liver-like lipid metabolic genes/pathways [14]. About 15 of these genes were also found enriched in our oenocyte translatome analysis (e.g., *Cpr, Cat, spidey, FarO*) (Additional file 2: List 16). About 463 human liver-enriched genes were retrieved from the Human Protein Atlas [44] (Additional file 2: List 15).

Interestingly, there was very little overlap between oenocyte-enriched and fat body-enriched genes, suggesting that adult fat body and oenocytes may regulate distinct biological processes (Additional file 1: Figure S3A, Additional file 2: List 14). Gene ontology analysis revealed that fat body mainly expressed genes in carboxylic acid and amino acid metabolism, whereas oenocytes were enriched with genes in pathways like fatty acid biosynthesis, fatty acid elongation, proteasome-mediated protein catabolism, xenobiotic metabolism, ketone body metabolism, and peroxisome (Table 1, Additional file 3: List 14–15). Furthermore, we found that two innate immunity pathways, Toll and Imd (Immune deficiency), were differentially enriched in fat body and oenocytes (Additional file 1: Figure S3B, Additional file 2: List 12–13). Several genes in Imd pathway (*PRGP-LC, PRGP-LB, Dredd*) were enriched in oenocytes, whereas fat body were enriched with genes in Toll pathway (*Tl, PGRP-SA, GNBP3, modSP*) (Additional file 1: Figure S3B). Additionally, most of the antimicrobial peptides (AMPs) were enriched in oenocytes, but not in fat body (Additional file 1: Figure S3B).

**Fig. 6:**
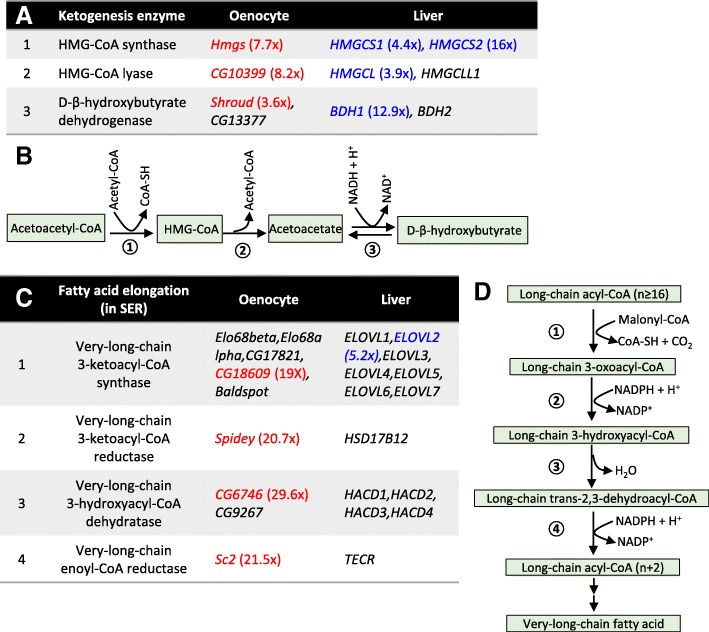
Ketogenesis and fatty acid elongation are enriched in both oenocytes and liver. **a** List of ketogenesis genes that are enriched in both oenocytes and liver. **b** Schematic diagram showing ketogenesis pathway. **c** List of genes in microsomal fatty acid elongation pathway that are enriched in both oenocytes and liver. **d** Schematic diagram showing microsomal fatty acid elongation pathway (in smooth ER). Oenocyte-enriched genes are highlighted in red. Liver-enriched genes are highlighted in blue

When comparing gene expression data between oenocyte and liver, we found that several pathways were specifically enriched in both liver and oenocytes, such as long-chain fatty acid metabolism, peroxisome, and xenobiotic metabolism (Table 1, Additional file 3: List 14–16). A close look at the enriched genes shared between oenocytes and liver revealed that *HMG-CoA synthase* (*Hmgs* in fly and *HMGCS1/2* in human), the key enzyme involved in ketogenesis and production of β-hydroxy-β-methylglutaryl-CoA (HMG-CoA), was highly expressed in both oenocytes and liver (Fig. 6a and b). Additionally, two other ketogenesis genes were also highly expressed in both oenocytes and liver. They are *HMG-CoA lyase* (*CG10399* in fly and *HMGCL* in human) and *D-β-hydroxybutyrate dehydrogenase* (*shroud* in fly and *BDH1* in human) (Fig. 6a and b). Ketogenesis is primarily activated in mammalian liver, especially during fasting. These results suggest that oenocyte may be the fly tissue regulating ketogenesis similar to mammalian liver.

Microsomal fatty acid elongation and the synthesis of very-long-chain fatty acid (VLCFA) were also enriched in both oenocytes and liver (Fig. 6c and d). Liver and oenocytes were enriched for very-long-chain 3-ketoacyl-CoA synthase (*ELOVL2* in human and *CG18609* in fly), which catalyzes the first step of VLCFA synthesis in smooth endoplasmic reticulum (smooth ER). Oenocytes also showed high expression of three other key enzymes in this process (*spidey, CG6746, Sc2*) (Fig. 6c and d). The enrichment of fatty acid elongation factors in oenocytes aligns well with previously known oenocyte function in the biosynthesis of VLCFA and hydrocarbons [21, 25]. Notably, several key players involved in the production of cuticular hydrocarbons were enriched in adult oenocytes, including cytochrome P450 *Cyp4g1* (3.4-fold) and its obligatory redox partner, cytochrome P450 reductase *Cpr* (5.7-fold), as well as five peroxisome-localized fatty acyl-CoA reductases (FAR) (*FarO, CG13091, CG14893, CG17562, and CG4020*) (Additional file 2: List 11–12). In particular, *FarO* was 123-fold enriched in oenocytes, while *CG13091* was 243-fold enriched (Additional file 1: Figure S4).

Additionally, many oenocyte- and liver-enriched genes belong to peroxisome pathway, especially peroxisomal beta-oxidation (*CG17597, CG9577* in oenocytes, *ACOX2, BAAT, EHHADH, ACAA1, SLC27A2, ACSL1, PECR* in liver) (Additional file 1: Figure S4). Genes involved in ROS metabolism (e.g., *Cat, Sod1*) were also enriched in both oenocytes and liver (Additional file 1: Figure S4). Lastly, we found that fibroblast growth factor 21 (*bnl* in fly and *FGF21* in human), a key hormonal factor that regulates glucose homeostasis, was enriched in both oenocytes and liver (Additional file 2: List 12&15). Taken together, our translatome analysis suggests that oenocytes and fat body regulate distinct processes, and oenocytes may participate several liver-like functions (e.g., ketogenesis, and long-chain fatty acid metabolism).

### Conservation in age-regulated signaling pathways between oenocytes and liver

**Fig. 7:**
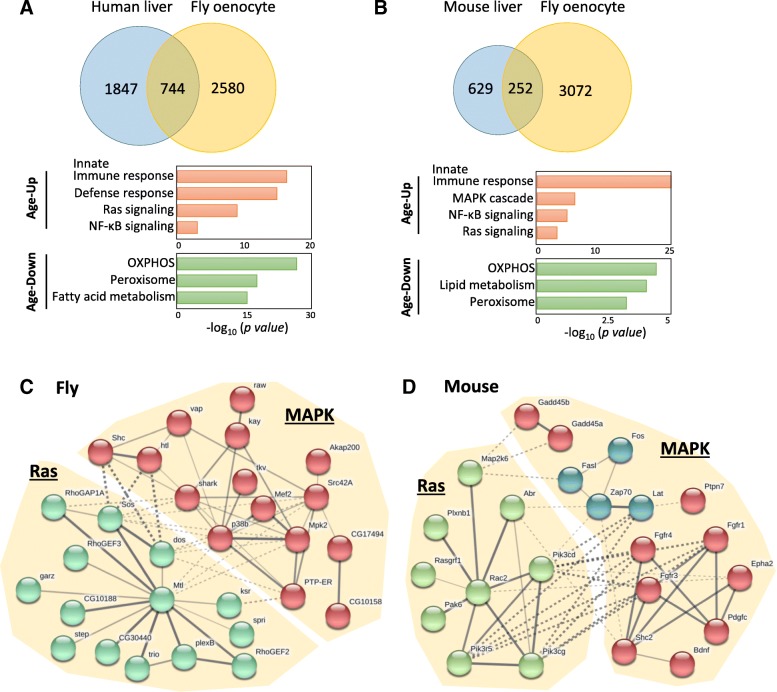
Conservation of age-related translational and transcriptional changes between oenocytes and liver. **a** Venn diagram comparing genes/proteins differentially expressed in aged human liver and aged fly oenocytes. GO terms for age-regulated proteins in human liver were shown in the lower panel. **b** Venn diagram comparing genes differentially expressed in aged mouse liver and aged fly oenocytes. GO terms for age-regulated genes in mouse liver were shown in the lower panel. OXPHOS: oxidative phosphorylation. **c** and **d** List of all genes in Ras/MAPK signaling pathway that were down-regulated in aged fly oenocytes and mouse liver. Protein network was generated using STRING (with *kmeans clustering* option). Continuum edge indicates a high confidence score. Dashed edge indicates a low confidence score

Since our analyses suggest that *Drosophila* oenocytes may perform liver-like functions, we wonder if oenocyte and liver exhibit similar translational changes during aging. To test this, we compared our oenocyte RiboTag data with recent proteomic analysis of aging human liver [13], and transcriptomic analysis of aging mouse liver [10]. We first searched for fly orthologues of human/mouse liver genes using *Drosophila* Integrative Ortholog Prediction Tool (DIOPT) [45]. Out of 3063 proteins differentially expressed in aging human liver, 2081 of them have putative orthologues in *Drosophila* genome (DIOPT rank is moderate or high), corresponding to 2214 *Drosophila* genes (Fig. 7a). About 35% of these *Drosophila* orthologues (774 out of 2214) also showed differential expression during oenocyte aging (Additional file 2: List 17–18). Similarly, 735 of out of 1052 differentially expressed genes in aging mouse liver have putative *Drosophila* orthologues (corresponding to 881 *Drosophila* genes) (Fig. 7b). About 30% of these *Drosophila* orthologues (252 out of 881) are differential expressed in aging oenocytes (Additional file 2: List 19–20).

Gene ontology analysis revealed that several age-regulated biological processes and signaling pathways were shared between mammalian liver and fly oenocytes (Fig. 7a-b) (Additional file 3: List 17–22). Genes up-regulated in both aged oenocytes and liver were enriched in pathways like innate immune response, Mitogen-activated protein kinase (MAPK) (e.g., p38b, kay, shark), Ras signaling (e.g., plexB), and Toll/NF-κB (e.g., Tl). On the other hand, commonly down-regulated genes in aging liver and oenocytes were found in oxidative phosphorylation (e.g., SdhC), fatty acid metabolism (e.g., Thiolase, whd), and peroxisome pathways (e.g., Catalase, ScpX). Using STRING protein network analysis, we found that large number of Ras/MAPK signaling components were up-regulated under both oenocyte and liver aging (Fig. 7c-d), suggesting that age-dependent dysregulation of these pathways are conserved between fly and mammal.

Lastly, we examined age-related gene expression changes between oenocytes and several other fly tissues, such as fat body, midgut, and heart. The age-related transcriptional profiles in these fly tissues were obtained from recent transcriptome studies (currently no translatomes for these tissues are available) [46–48] (Additional file 2: List 21–22). We recognize that there are known caveats for the transcriptome and translatome comparison, due to large variations in translation efficiency among genes. Pathway analysis (using STRING) revealed tissue-specific transcriptional profiles during fly aging (Additional file 1: Figure S5). For example, genes that were differentially expressed in aged oenocytes are enriched for proteasome and ribosome-related functions, while aged fat body showed transcriptional changes in aminoglycan metabolism, chitin metabolism, and detoxification. In aging heart, immune response, glycolysis and gluconeogenesis were enriched. And ion transport, DNA replication, and fatty acid degradation were altered in aging midgut. Taken together, aged oenocytes share similar expression profiles with aging liver, while they also exhibit unique features compared to other fly tissues.

## Discussion

Oenocytes are poorly studied yet important cells in insects [22, 23]. Although previous studies show that oenocytes play a crucial role in lipid metabolism (e.g., synthesis of cuticular hydrocarbon and pheromone), many other oenocyte-regulated physiological functions remain to be determined. Among the uncharacterized functions, we know very little about oenocyte aging and the role of oenocytes in aging regulation. To address these issues, we performed RiboTag sequencing to characterize *Drosophila* oenocyte translatome under aging and oxidative stress. We show that both aging and paraquat up-regulated DNA repair pathway, while down-regulating immune response and fatty acid elongation. In addition, aged oenocytes were associated with impaired peroxisome, mitochondrial, proteasome, and cytochrome P450 pathways. Our RiboTag sequencing also revealed many shared tissue-specific pathways and age-related translational changes between fly oenocytes and mammalian liver, highlighting evolutionarily conserved mechanisms underlying oenocyte and liver aging and potential functional homologies between the two tissues.

### Oenocyte-specific expressed genes are involved in both insect-specific and conserved liver-like functions

Previous functional and histological analyses showed that oenocytes contain large amounts of smooth ER and acidophilic cytoplasm (high protein and lipid contents) [49, 50], which is consistent with their roles in lipid synthesis and processing, especially the production of VLCFA and hydrocarbon [21, 25, 51, 52]. Interestingly, *Drosophila* oenocytes uptake and process fatty acids that are released from the storage tissue fat body during food deprivation [15]. The coordination between fat body and oenocytes in mobilizing lipid storage during fasting is quite similar to the adipose-liver axis in mammals. Besides lipid metabolism, many other oenocyte-associated functions (e.g., detoxification and ecdysteroid biosynthesis) have not yet been thoroughly examined at the molecular level. It is unclear whether some of these functions are also conserved liver-like functions, or they are merely insect-specific roles.

To better understand oenocyte function, we conducted oenocyte-specific translatome profiling in adult *Drosophila* and identified 423 genes that were highly expressed in oenocytes (at least 5-fold higher than whole body expression). These genes were enriched in pathways like fatty acid elongation, proteasome-mediated protein catabolism, xenobiotic metabolism, ketogenesis, and peroxisome pathways. There was only a small overlap between oenocyte-enriched and fat body-enriched genes, suggesting that the two tissues regulate distinct functions in *Drosophila*. Comparing to the genes and pathways enriched in human liver, we found that oenocytes shared several biological processes with liver, such as ketogenesis, peroxisomal beta-oxidation, ROS metabolism, long-chain fatty acid metabolism, and xenobiotic metabolism. This is consistent with a previous study showing that *Drosophila* oenocytes expressed high levels of lipid metabolic genes similar to those of mammalian liver [15]. One enriched pathway in *Drosophila* oenocytes that was not observed in the previous study is the ketogenesis pathway. It is well-known that ketone bodies (acetoacetate, β-hydroxybutyrate, and acetone) are primarily produced by liver when glucose is not available as fuel source [53]. Ketogenesis in insects, however, is not well studied. Ketone bodies have been detected in hemolymph, fat body, and thoracic muscle of adult desert locust and cockroach [54–56]. It is speculated that ketone bodies are produced in fat body according to the ex vivo tissue culture assay in locust [55]. However, fat body (along with many other tissues) can also oxidize ketone bodies, which is quite different from mammals where the ketogenesis tissue liver cannot oxidize ketone [55]. It might be possible that in previous ex vivo tissue culture studies, the ketone production came from a contaminated tissue (like oenocytes), rather than fat body. Based on our oenocyte translatome analysis, most of the ketogenesis genes are highly expressed in oenocytes, but not in fat body. Our data suggest that oenocytes are likely the major ketogenesis tissue. A careful function and genetic analysis, such as cell ablation or tissue-specific gene silencing, will need to be performed to examine whether oenocytes are responsible for ketogenesis in *Drosophila* and in other insect species.

Insect hydrocarbons serve as important waterproofing components, and species- and sex-specific recognition signals. The biosynthesis of hydrocarbons are involved in fatty acid elongation, desaturation, reduction, and oxidative decarbonylation [57]. Our oenocyte translatome analysis revealed an enrichment of genes in microsomal fatty acid elongation system, such as *CG18609, spidey, CG6746,* and *Sc2*. This is consistent with oenocyte’s role in hydrocarbon production and its abundant smooth ER content. In microsomal fatty acid elongation system, *spidey* (also known as *Kar*) encodes for the only very-long-chain 3-ketoacyl-CoA reductase in *Drosophila* genome, and it has been implicated in oenocyte VLCFA synthesis and waterproof of the trachea system [52], as well as the production of cuticular hydrocarbon, ecdysteroid metabolism, and oenocyte maturation [25, 58]. Final two steps of hydrocarbon production in insects are very-long-chain fatty acyl-CoA to aldehydes conversion by FAR and aldehyde oxidative decarbonylation by Cyp4g1 and Cpr [22, 39]. Our translatome analysis showed that five different *FARs* (including *FarO*), *Cyp4g1*, and *Cpr* are highly expressed in adult oenocytes. The large number of FARs expressed in adult oenocytes suggests that aldehyde-forming FARs may be responsible for the production of a variety of hydrocarbons in oenocytes, and each FAR can catalyze a unique set of very-long-chain fatty acyl-CoA esters that vary in saturation status and chain length.

In adult insects (especially in females), ovary is the major tissue for ecdysteroid biosynthesis [59, 60]. It remains to be determined whether other adult tissues are also capable to synthesize ecdysteroids. Interestingly, we found two Halloween genes (*phantom* and *shadow*) that are highly expressed in adult oenocytes, suggesting that oenocytes may participate in ecdysteroid synthesis in adult females. Our findings are consistent with an early study showing that abdominal oenocytes dissected from *Tenebrio molitor* larvae can synthesize 20-Hydroxyecdysone (β-ecdysone) [61]. Several recent studies also detected the expression of Halloween genes in adult tissues other than ovaries, such as brain [62], fat body, muscle, and Malpighian tubule [63]. To functionally verify the role of adult oenocytes in ecdysteroid biosynthesis, direct measurement of ecdysteroid production is needed when Halloween genes are specifically knocked down in oenocytes.

### Impaired peroxisome pathway and fatty acid beta-oxidation are the hallmarks of oenocyte aging

Our translatome analysis identified large number of genes (1092 up-regulated and 2232 down-regulated) that were differentially expressed between young and middle ages, suggesting that large cellular and molecular alterations can be observed in oenocytes at the middle age. Some of these changes are consistent with previous aging transcriptome analysis in *Drosophila* [32, 33, 64], such as up-regulation of DNA repair and down-regulation of oxidative phosphorylation. On the other hand, oenocyte aging was specifically associated with the dysregulation of several other pathways, such as down-regulation of peroxisome and fatty acid metabolism pathways. Peroxisomes are important subcellular organelles that participate in a variety of metabolic pathways, including alpha-oxidation of phytanic acids, beta-oxidation of VLCFA, ether phospholipid synthesis (e.g., plasmalogen biosynthesis), ROS and hydrogen peroxide metabolism, glyoxylate metabolism, catabolism of amino acids and purine [65]. There are about 16 peroxisome biogenesis genes (also known as peroxin, or PEX) in *Drosophila* that are responsible for peroxisome membrane assembly (Pex3, Pex6, Pex9), matrix enzyme import and receptor recycling (Pex5, Pex7, Pex13, Pex14, Pex2, Pex10, Pex12, Pex1, Pex6), and peroxisome proliferation (Pex11) [66]. Mutation in many peroxin genes leads to various forms of peroxisome biogenesis disorder (PBD), also known as Zellweger syndrome (ZS) in human [65]. Our data revealed that aging and PQ-induced oxidative stress decreased the expression of most of the peroxisome biogenesis and protein import genes, which may lead to reduced peroxisome function, including hydrogen peroxide metabolism. Decreased expression of receptor protein Pex5 and reduced peroxisomal enzyme import were previously observed in aged *C. elegans* [40] and during human fibroblast senescence [67]. Among many key peroxisomal enzymes, the importing of antioxidant catalase was significantly affected during fibroblast senescence, which led to accumulation of hydrogen peroxide and further disruption of peroxisome import [67]. Similar to early studies in aging rat liver [68–70], we found that the expression of many peroxisomal antioxidant enzymes (e.g., *Cat, SOD1, Prx5*) decreased in aged oenocytes. The combined dysregulation of peroxisomal gene expression and protein import may attribute to elevated toxic reactive oxygen species, and impaired oenocyte functions. Furthermore, generation of excess peroxisomal ROS could disrupt mitochondria redox balance, leading to mitochondrial dysfunction and tissue aging [71].

Impaired peroxisome biogenesis/protein import during aging not only contributes to reduced antioxidant capacity and elevated ROS levels, but also dysregulation of other peroxisomal functions. Besides ROS metabolism, our translatome analysis revealed that genes involved in peroxisomal beta-oxidation and ether phospholipid were down-regulated under oenocyte aging. This is consistent with previous studies showing that peroxisomal beta-oxidation activity decreased in old mouse liver [72]. Peroxisome has been shown to coordinate with mitochondrial fission/fusion pathway to regulate cellular fatty acid oxidation [73], a major metabolic process dysregulated during mouse aging [74]. Although the metabolic reactions for fatty acid beta-oxidation are similar in mitochondria and peroxisome, a set of fatty acid substrates can only be processed by peroxisomes, such as VLCFA, pristanic acid, di- and trihydroxycholestanoic acid (DHCA and THCA), long-chain dicarboxylic acids, certain polyunsaturated fatty acids [65, 75]. Mutation of peroxisome fatty acid transporter ABCD1 impaired peroxisomal beta-oxidation and caused to accumulation of VLCFAs and neuroinflammation, which is associated with X-link neurodegenerative disease adrenoleukodystrophy (ALD) [76, 77]. Mouse homozygous mutants of ACOX, which catalyzes the first step of peroxisomal beta-oxidation, also showed accumulation of VLCFA and development of microvesicular fatty liver. Although the expression of two *Drosophila* ACOX genes were not significantly altered during oenocyte aging, ScpX (peroxisomal thiolase) was significantly down-regulated. Mice with ScpX mutation showed defects in peroxisome proliferation, hypolipidemia, motor and peripheral neuropathy, as well as impaired catabolism of methyl-branched fatty acids [78]. In addition, reduced peroxisome function can disrupt lipid homeostasis and lipid composition, which could lead to compromised immune response [79, 80].

### Conservation between oenocytes and liver aging

The comparison of aging translatome/transcriptome between fly oenocytes and mammalian liver revealed many shared pathways between the two tissues. Among these conserved pathways, MAPK and Ras signaling pathways were significantly up-regulated in both aged oenocytes and liver. MAPK signaling is one of the major regulatory pathways involved in stress responses (e.g., oxidative stress). The typical MAPK pathway includes three branches: c-Jun N-terminal kinase (JNK), p38/MAPK, and extracellular signal-regulated kinase (ERK). Previous studies show that all three MAPK cascades are elevated under aging, probably due to increased oxidative stress [81, 82]. Dysregulated MAPK signaling has been implicated in cancer and neurodegenerative diseases such as Alzheimer’s disease, Parkinson’s disease, and amyotrophic lateral sclerosis (reviewed in [83]). In model organisms (e.g., *Drosophila* and *C. elegans*), activation of JNK and p38/MAPK extended lifespan and improved tissue functions in late life [84–86]. Among many MAPK components identified in our analysis, the activator protein 1 (AP-1) subunit, *Drosophila* Kay and its mouse orthologue c-Fos, were found significantly induced under aging. Both Kay and c-Fos are basic leucine zipper transcription factors that mediate MAPK signaling (especially JNK pathway) to regulate cell proliferation, tissue regeneration, stress tolerance [87, 88]. Since JNK signaling is the key regulator for the maintenance of tissue homeostasis in response to intrinsic and extrinsic stresses (e.g., UV irradiation, ROS, DNA damage, inflammatory cytokines, infection), the induction of Kay/c-Fos indicates an up-regulation of JNK signaling (and other MAPK pathways), as well as an elevated cellular stress responses in both aged oenocytes and liver. In addition, Ras small GTPase pathway, the upstream regulator of MAPK kinase cascades, was also up-regulated during oenocyte and liver aging. The direct role of Ras signaling pathway in longevity regulation has been previously demonstrated in several model organisms [89–92]. Further studies on Ras/MAPK signaling are needed to advance our understanding on the specific contributions of these pathways in oenocyte and liver aging. Nevertheless, the up-regulation of Ras/MAPK signaling pathways can be used as an important molecular signature and biomarker for oenocyte and liver aging.

## Conclusion

Using RiboTag sequencing, we characterized the first oenocyte translatome profiles in *Drosophila*. Our analysis uncovered many previously unexplored oenocyte-specific molecular pathways, especially those associated with oxidative stress and aging. Some of these pathways were enriched in both fly oenocytes and mammalian liver, suggesting a functional homology between the two tissues. We believe that the analysis of oenocyte translatome will contribute significantly to our understanding of oenocyte biology, as well as the molecular mechanisms for its role in stress response and aging regulation.

## Methods

### Fly strains, aging and paraquat treatment

Flies are raised in 12 h:12 h light:dark cycle at 25 °C, 60% relative humidity on agar-based diet with 0.8% cornmeal, 10% sugar, and 2.5% yeast (unless otherwise noted). Fly strains used in the present study include: w*; PromE-Gal4 (also known as *Desat1-GAL4.E800*) (Bloomington #65405) [21], *PromE-Gal4; UAS-CD8::GFP* (a gift from Alex Gould). *UAS-RpL13A-FLAG* transgenic fly lines were generated through germ line transformation of a standard pUAST vector containing full-length RpL13A fused with FLAG. The oenocyte-specific expression of *RpL13A* is under the control of an upstream activating sequence (UAS) upon GAL4 protein binding. To age flies, females were collected 2 days after eclosion, and 20 females per vial were maintained at 25 °C and transferred to fresh food every 2–3 days. Two ages were tested, young (10-day-old) and middle age (30-day-old). For paraquat treatment, flies were fed on fly food containing 10 mM of paraquat (at the food surface) for 24 h prior to each assay.

### Dihydroethidium (DHE) staining

Young and aged flies were fed on normal food or paraquat (10 mM) for 24 h prior to the staining with dihydroethidium (Calbiochem, Burlington, MA, USA. Catalog number: 38483–26-0). DHE staining was performed as previously described [93]. Briefly, fly abdomen was dissected out (fat body removed) and incubated with 30 μM of DHE in Schneider’s *Drosophila* Medium (ThermoFisher Scientific, Catalog number: 21720–024) for 5 min in a dark chamber on an orbital shaker. After additional 5 min incubation with 1 μg/mL of Hoechst 33342 (ImmunoChemistry Technologies, Bloomington, MN, USA. Catalog number: 639), fly abdomen was mounted with 50% glycerol in PBS. DHE staining was visualized with Olympus BX51WI upright epifluorescence microscopy.

### Oenocyte RiboTag

Female progeny from the crosses between *PromE-gal4* and *UAS-RpL13A-FLAG* were collected 2 days after eclosion. Four different experimental groups were tested: 1). 10-day-old females fed on normal food (H2O-Young); 2). 10-day-old females treated with 10 mM of paraquat for 24 h (PQ-Young); 3). 30-day-old females fed on normal food (H2O-Aged); 4). 30-day-old females treated with 10 mM of paraquat for 24 h (PQ-Aged). Three biological replicates (200 females per replicate) were performed for each group. Female flies were used in the present study, because *PromE-gal4* drives expression in testis (additional to oenocytes) in male flies [21].

RiboTag was performed following the protocol from McKnight Lab [30]. Briefly, flies were first frozen and ground in nitrogen liquid. The fly powder was then further homogenized in a Dounce tissue grinder containing 5 mL of homogenization buffer (50 mM Tris-HCl, pH 7.4, 100 mM KCl, 12 mM MgCl2, 1 mM DTT, 1% NP-40, 400 units/ml RNAsin RNase inhibitor, 100 μg/ml of cycloheximide, 1 mg/ml heparin, and Protease inhibitors). After centrifuging the homogenate at 10,000 rpm for 10 min, the supernatant was first pre-cleaned using SureBeads™ Protein G Magnetic Beads (Bio Rad, Hercules, CA, USA. Catalog number: 161–4023), and then incubated with 15 μl of anti-FLAG antibody (Sigma-Aldrich, St. Louis, MO, USA. Catalog number: F1804) for about 19 h at 4 °C. The antibody/lysate mixture was then incubated with 100 μl of SureBeads for 3 h at 4 °C. Ribosome-bound RNA was extracted and purified using RNeasy Plus Micro Kit (Qiagen, Hilden, Germany. Catalog number: 74034).

### Translatome library construction and high-throughput sequencing (RNA-Seq)

RNA-Seq libraries were constructed using 300 ng of total RNA and NEBNext Ultra Directional RNA Library Prep Kit for Illumina (New England Biolabs (NEB), Ipswich, MA, USA. Catalog number: E7420). RNA concentrations were measured using Qubit RNA BR Assay Kit (Thermo Fisher Scientific, Waltham, MA, USA Catalog number: Q10210). Poly(A) mRNA was isolated using NEBNext Oligo d(T)_25_ beads and fragmented into 200 nt in size. After first strand and second strand cDNA synthesis, each cDNA library was ligated with a NEBNext adaptor and barcoded with an adaptor-specific index. Twelve libraries were pooled in equal concentrations, and sequenced using Illumina HiSeq 3000 platform (single-end, 50 bp reads format).

### RNA-Seq data processing and differential expression analysis

The RNA-Seq data processing was performed on Galaxy, an open source, web-based bioinformatics platform (https://usegalaxy.org) [94]. FastQC was first performed to check the sequencing read quality. Then the raw reads were mapped to *D. melanogaster* genome (BDGP Release 6 + ISO1 MT/dm6) using Tophat2 v2.1.0 [95]. Transcripts were reconstructed using Cufflinks v2.2.1 with bias correction. Cuffmerge (http://cole-trapnell-lab.github.io/cufflinks/) was used to merge together 12 Cufflinks assemblies to produce a GTF file for further differential expression analysis with Cuffdiff v2.2.1.3 [96]. After normalization, differentially expressed protein-coding transcripts were obtained using following cut-off values, false discovery rate (FDR) ≤ 0.05 and fold-change ≥2. Non-coding gene and low expressed genes (FPKM< 0.01) were excluded from the analysis. RNA-Seq read files have been deposited to NCBI ‘s Gene Expression Omnibus (GEO) (Accession # GSE112146). To review GEO files: Go to https://www.ncbi.nlm.nih.gov/geo/query/acc.cgi?acc=GSE112146.

### Principal component analysis (PCA), heatmap and expression correlation plot

PCA graph was generated using plotPCA function of R package DESeq2 [97]. Heatmaps and hierarchy clustering analysis were generated using heatmap.2 function of R package gplots. (https://cran.r-project.org/web/packages/gplots). Expression data was log2 transformed and all reads were added by a pseudo-value 1. The expression correlation plots were plotted using R package ggplot2 (https://cran.r-project.org/web/packages/ggplot2).

### Oenocyte-enriched genes and tissue-specific aging translatome/transcriptome analysis

Oenocyte-enriched genes were identified by comparing our oenocyte RiboTag data (H2O Young group) to the whole body transcriptome profiles from previous studies (two wild-type backgrounds: *w*^*1118*^*:* GSM2647344, GSM2647345, GSM2647345. *yw:* GSM694258, GSM694259). The sequencing reads with FPKM ≥0.01 were normalized by quantile normalization function using preprocessCore package. (https://www.bioconductor.org/packages/release/bioc/html/preprocessCore.html). Oenocyte-enriched genes were defined as those with 5-fold higher FPKM in oenocytes comparing to whole body. Fat body-enriched genes were obtained similarly by comparing the expression values between adult fat body and whole body (data retrieved from FlyAtlas).

The lists of differentially expressed genes in multiple fly tissues were extracted from previous transcriptome analyses, heart [46], posterior midgut [48], fat body [47]. Venn diagram analysis (http://bioinformatics.psb.ugent.be/webtools/Venn/) was performed to identify overlapping genes between different tissues.

### Gene set enrichment analysis (GSEA)

For GSEA analysis, a complete set of 136 KEGG pathways in *Drosophila* were downloaded from KEGG. Text were trimmed and organized using Java script. Quantile normalized FPKM values for each group were used as input for parametric analysis, and organized as suggested by GSEA tutorial site (GSEA, http://software.broadinstitute.org/gsea/doc/GSEAUserGuideFrame.html) [98]. Collapse dataset to gene symbols was set to false. Permutation type was set to gene set; enrichment statistic used as weighted analysis; metric for ranking genes was set to Signal to Noise.

### Gene ontology and pathway analysis

Functional annotation analysis of differentially expressed genes was performed using STRING. GO terms (Biological Process, Molecular Function, Cellular Component), KEGG pathway, INTERPRO Protein Domains and Features, were retrieved from the analysis. To build Ras/MAPK protein network in STRING, “kmeans clustering” option was used and number of clusters was set to 2 or 3.

### Quantitative real-time polymerase chain reaction (qRT-PCR)

qRT-PCR was performed using Quantstudio 3 Real-Time PCR system and SYBR green master mixture (Thermo Fisher Scientific, Waltham, MA, USA Catalog number: A25778). To determine the most stable housekeeping gene, the Ct values for four housekeeping genes were examined in all 12 cDNA samples obtained from different treatments. Using an Excel-based tool, *Bestkeeper* [99], we confirmed that *Gapdh1* is the least-variable housekeeping gene across samples (Additional file 4). All gene expression levels were normalized to *Gapdh1* by the method of comparative Ct [100]. Mean and standard errors for each gene were obtained from the averages of three biological replicates, with one or two technical repeats. Primer sequences are available in Additional file 5.

### Statistical analysis

GraphPad Prism (GraphPad Software, La Jolla, CA, USA) was used for statistical analysis. To compare the mean value of treatment groups versus that of control, either student t-test or one-way ANOVA was performed using Dunnett’s test for multiple comparison.

## Additional files

Additional file 1: **Figure S1.** Age-dependent PromE-gal4 expression pattern. (A-B) Fluorescent image PromE-Gal4; UAS-CD8::GFP female flies at two ages: Young (10-day-old), Aged (30-day-old). Scale bar: 50 μm. (C) Quantification of GFP intensity from Panel (A&B). Student t-test (ns = not significant). *N*=9. **Figure S2.** Two ecdysteroid biosynthesis genes highly express in oenocytes. Schematic diagram showing ecdysteroid hormone metabolism pathway. Two Halloween genes, phantom and shadow, highly expressed in adult female oenocytes (Highlightedinred). **Figure S3.** Genes in innate immunity pathway highly express in oenocytes. (A) Genes enriched in oenocytes and fat body show less overlap. (B) Genes in Imd pathway were enriched in oenocytes, while fat body were enriched with genes in Toll pathway (Red arrows denote for age-induced genes. Blue arrows denote for age-repressed gene.). **Figure S4.** Peroxisome pathways are enriched in both oenocytes and liver. List of peroxisome genes that are enriched in both oenocytes and liver. **Figure S5.** (A) Venn diagram showing the overlap of differentially expressed genes in aged oenocytes, fat body, heart, and midgut. (B) GO terms enriched in aged oenocytes, fat body, heart, and midgut. (PDF 452 kb)

Additional file 2: All gene lists. (XLSX 3055 kb)

Additional file 3: All GO term tables. (XLSX 1836 kb)

Additional file 4: Housekeeping gene analysis using *Bestkeeper*. (XLSX 11 kb)

Additional file 5: qRT-PCR Primer list. (XLSX 10 kb)

## Abbreviations

AMP: Antimicrobial peptide
Cat: Catalase
CYP: Cytochrome P450
Desat1: Desaturase 1
DGE: Differential gene expression
DHAPAT: Dihydroxyacetone phosphate acyltransferase
DIOPT: Integrative Ortholog Prediction Tool
FAR: Fatty acyl-CoA reductase
FPKM: Fragments per kilobase million
GO: Gene ontology
GSEA: Gene set enrichment analysis
GST: Glutathione S-transferase
Imd: Immune deficiency
KEGG: Kyoto Encyclopedia of Genes and Genomes
MAPK: Mitogen-activated protein kinase
Mgstl: Microsomal glutathione S-transferase
NAFLD: Non-alcoholic fatty liver disease
NF-κB: Activation of nuclear factor-κB
PCA: Principal component analysis
PQ: Paraquat
Prx5: Peroxiredoxin 5
Sod1: Superoxide dismutase 1
VLCFA: Very-long-chain fatty acid
