# Supplementary material for: RiboTag translatomic profiling of Drosophila oenocytes under aging and induced oxidative stress
Source: BMC Genomics. 2019 Jan 16;20:50. doi: 10.1186/s12864-018-5404-4 (PMC6335716; doi:10.1186/s12864-018-5404-4)
Supplement: Supplementary file 1 — Figure S1. Age-dependent PromE-gal4 expression pattern. (A-B) Fluorescent image PromE-Gal4; UAS-CD8::GFP female flies at two ages: Young (10-day-old), Aged (30-day-old). Scale bar: 50 μm. (C) Quantification of GFP intensity from Panel (A&B). Student t-test (ns = not significant). N=9. Figure S2. Two ecdysteroid biosynthesis genes highly express in oenocytes. Schematic diagram showing ecdysteroid hormone metabolism pathway. Two Halloween genes, phantom and shadow, highly expressed in adult female oenocytes (Highlightedinred). Figure S3. Genes in innate immunity pathway highly express in oenocytes. (A) Genes enriched in oenocytes and fat body show less overlap. (B) Genes in Imd pathway were enriched in oenocytes, while fat body were enriched with genes in Toll pathway (Red arrows denote for age-induced genes. Blue arrows denote for age-repressed gene.). Figure S4. Peroxisome pathways are enriched in both oenocytes and liver. List of peroxisome genes that are enriched in both oenocytes and liver. Figure S5. (A) Venn diagram showing the overlap of differentially expressed genes in aged oenocytes, fat body, heart, and midgut. (B) GO terms enriched in aged oenocytes, fat body, heart, and midgut. (PDF 452 kb) [file 12864_2018_5404_MOESM1_ESM.pdf]

Figure S1

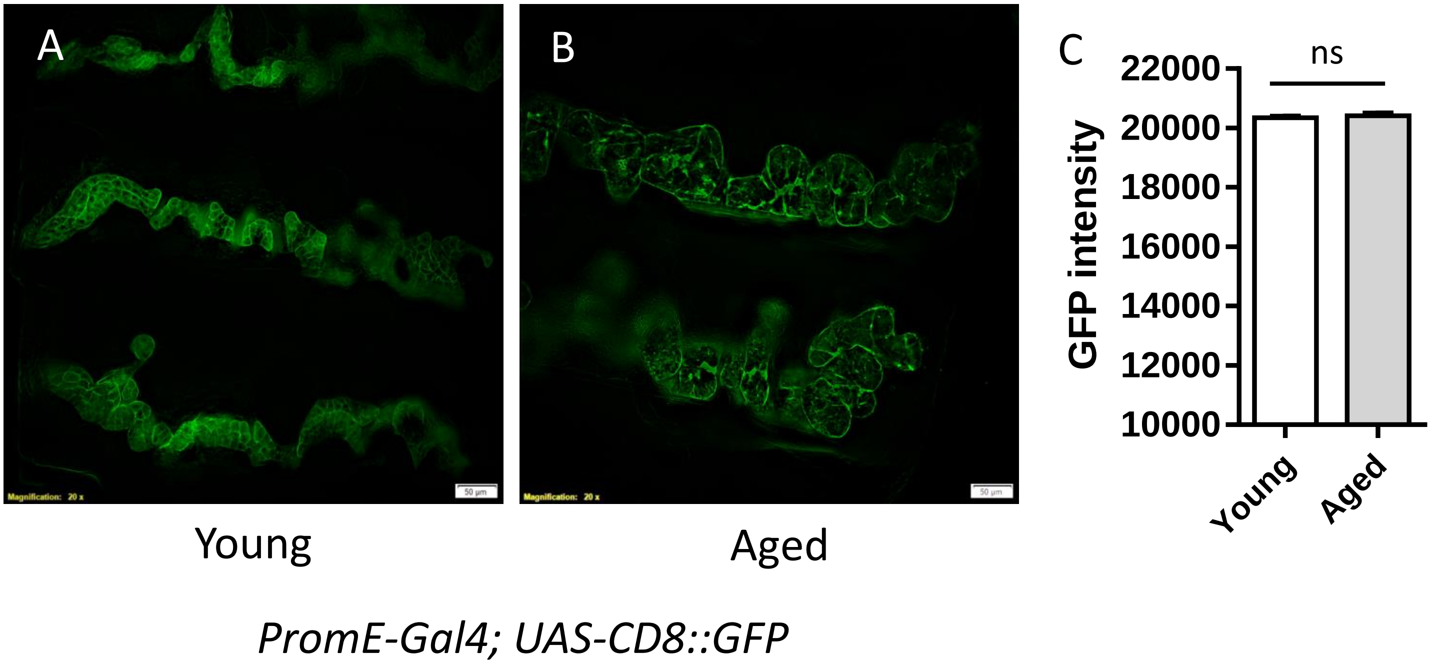

Figure S1. Age-dependent PromE-gal4 expression pattern. (A-B) Fluorescent image PromE-Gal4; UAS-CD8::GFP female flies at two ages: Young (10-day-old), Aged (30-day-old). Scale bar: 50 µm. (C) Quantification of GFP intensity from Panel (A&B). Student t-test (ns = not significant). N=9.

Figure S2

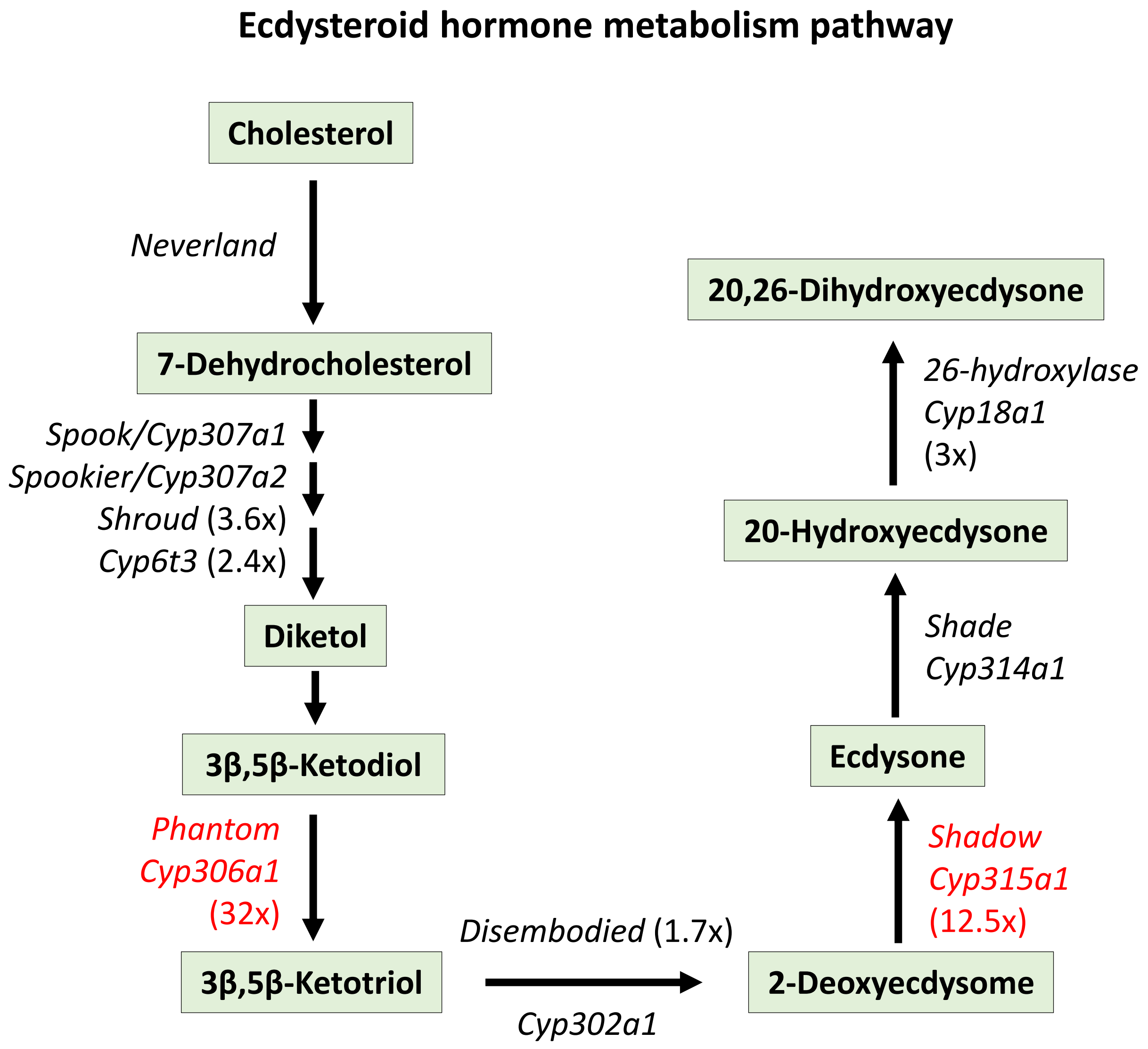

Figure S2. Two ecdysteroid biosynthesis genes highly express in oenocytes. Schematic diagram showing ecdysteroid hormone metabolism pathway. Two Halloween genes, phantom and shadow, highly expressed in adult female oenocytes (Highlighted in red).

Figure S3

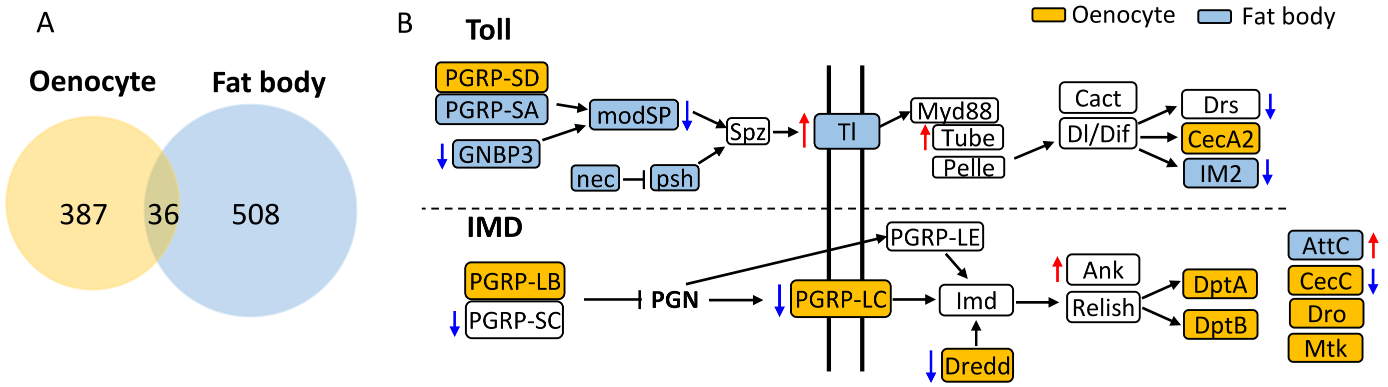

Figure S3. Genes in innate immunity pathway highly express in oenocytes. (A) Genes enriched in oenocytes and fat body show less overlap. (B) Genes in Imd pathway were enriched in oenocytes, while fat body were enriched with genes in Toll pathway (Red arrows denote for age-induced genes. Blue arrows denote for age-repressed gene.).

Figure S4

1. Peroxisome biogenesis and protein import

Oenocyte	Liver
Pex19 (7.4x)	PXMP2 (5.1x)

2. Peroxisome function

Fatty acid beta-oxidation

Oenocyte	Liver
SCPX/CG17597 (11.9x)<br>ECH/CG9577 (8.2x)	ACOX2 (7.5x)<br>BAAT (34.6x)<br>EHHADH (12x)<br>ACAA1 (6.3x)<br>SLC27A2 (9x)<br>ACSL1 (6.4x)<br>PECR (7.8x)

Ether phospholipid biosynthesis

Oenocyte	Liver
FarO (123x)<br>CG13091(243x)<br>CG14893 (21x)<br>CG17562 (40x)<br>CG4020 (51x)	None

ROS metabolism

Oenocyte	Liver
Cat (11.8x)<br>Sod1(18.7x)<br>Prx5 (6.4x)	CAT (5.2x)<br>SOD1 (3.3x)

Amino acid metabolism

Oenocyte	Liver
AGXT/Spat (2.2x)<br>HMGCL/CG10399 (8.2x)	AGXT (36.3x)<br>DAO (16.1x)<br>PIPOX (18.5x)<br>HAO1(36.8x)<br>HAO2 (15x)

Purine metabolism

Oenocyte	Liver
AOX1 (3.3x)	XDH (5.5x)

Retinol metabolism

Oenocyte	Liver
DHRS4/ CG10672 (16.3x)	None

Figure S4. Peroxisome pathways are enriched in both oenocytes and liver. List of peroxisome genes that are enriched in both oenocytes and liver.

Figure S5

A

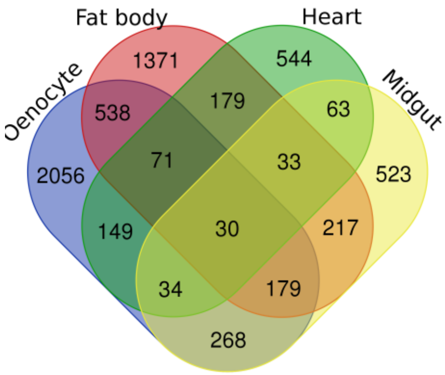

B

Oenocyte	Fat body	Heart	Midgut
Proteasome	Aminoglycan metabolism	Immune response	Ion transport
Ribosome	Chitin metabolic process	Glycolysis / Gluconeogenesis	DNA replication
Oxidative phosphorylation	Detoxification of inorganic compound	Oxidative phosphorylation	Fatty acid degradation

Figure S5. (A) Venn diagram showing the overlap of differentially expressed genes in aged oenocytes, fat body, heart, and midgut. (B) GO terms enriched in aged oenocytes, fat body, heart, and midgut.
